# Identification of 1-Butyl-Lysergic Acid Diethylamide (1B-LSD) in Seized Blotter Paper Using an Integrated Workflow of Analytical Techniques and Chemo-Informatics

**DOI:** 10.3390/molecules25030712

**Published:** 2020-02-07

**Authors:** Emmanouil Tsochatzis, Joao Alberto Lopes, Fabiano Reniero, Margaret Holland, Jenny Åberg, Claude Guillou

**Affiliations:** 1 European Commission, Joint Research Centre (JRC), I-20127 Ispra, Italy; Emmanouil.Tsochatzis@ec.europa.eu (E.T.); Fabiano.RENIERO@ec.europa.eu (F.R.); Margaret.HOLLAND@ec.europa.eu (M.H.); 2 Swedish Customs Laboratory, Box 6055, SE-171 06 Solna, Sweden; Jenny.ABERG@tullverket.se

**Keywords:** blotter paper sample, 1-butyl-lysergic acid diethylamide (1B-LSD), HR–MS/MS, NMR, GC–MS

## Abstract

The rapid dispersion of new psychoactive substances (NPS) presents challenges to customs services and analytical laboratories, which are involved in their detection and characterization. When the seized material is limited in quantity or of a complex nature, or when the target substance is present in very small amounts, the need to use advanced analytical techniques, efficient workflows and chemo-informatics tools is essential for the complete identification and elucidation of these substances. The current work describes the application of such a workflow in the analysis of a single blotter paper, seized by Swedish customs, that led to the identification of a lysergic acid diethylamide (LSD) derivative, 1-butyl-lysergic acid diethylamide (1B-LSD). Such blotter paper generally contains an amount in the range of 30–100 ug. This substance, which is closely related to 1-propionyl-lysergic acid diethylamide (1P-LSD), seems to have only recently reached the drug street market. Its identification was made possible by comprehensively combining gas chromatography with mass spectrometry detection (GC–MS), liquid chromatography coupled with high-resolution tandem MS (LC–HR-MS/MS), Orbitrap-MS and both 1D and 2D nuclear-magnetic-resonance (NMR) spectroscopy. All the obtained data have been managed, assessed, processed and evaluated using a chemo-informatics platform to produce the effective chemical and structural identification of 1B-LSD in the seized material.

## 1. Introduction

During the past decade, there has been a significant increase in the circulation of new psychoactive substances (NPS) within the EU. This evolution in the drugs market, and the speed at which new substances are being created, is a cause of great concern for regulatory bodies. In 2016, NPS were reported to the EU Early Warning System at a rate of one per week, and it is estimated that a similar trend continued during the following years [1]. At the EU level, two agencies have a particular interest in this field—the European Monitoring Center for Drugs and Drug Addiction (EMCDDA) and EUROPOL [1,2,3].

Customs authorities are responsible for controlling the flow of goods into the EU, acting as the first control and contact point for NPS. However, the majority of customs laboratories are not equipped for the analysis of these substances, as they normally lack the advanced analytical and chemo-informatics tools that enable the complete identification and characterization of any new and/or relatively unknown NPS. These tools and expertise are available at the European Commission’s Joint Research Centre (JRC), and, therefore, a collaborative project with the Customs Laboratories European Network (CLEN) has been established. Whenever an EU customs laboratory cannot completely identify an unknown seized substance/mixture, it is sent to the JRC for additional analysis. Since 2014, dozens of substances have been identified in the frame of this collaboration, including some which were previously unreported [3].

**Figure 1:**
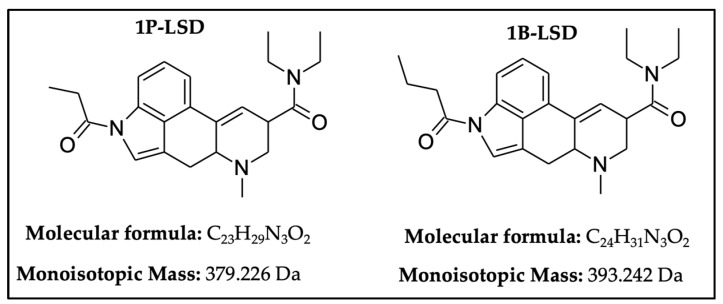
Chemical structures, molecular formulas and molecular masses (Da) of 1-butyl-lysergic acid diethylamide (1B-LSD) and 1-propionyl-lysergic acid diethylamide (1P-LSD).

One of the most well-known and characterized psychoactive substances is d-lysergic acid diethylamide (LSD), which is most commonly circulated through small pieces of paper called “blotter paper” and is widespread all over the world because of its strong hallucinogenic effect [4]. In recent years, structurally modified LSD-type NPS have been found on the market, as is reported in several scientific papers [5,6,7,8,9]. One of them, 1-propionyl-d-lysergic acid diethylamide (1P-LSD), was identified and characterized after extraction from a seized sample by applying ^1^H and ^13^C-NMR along with GC–MS and UHPLC-qTOF-MS analysis [5]. Another closely related LSD derivative, 1-butanoyl-d-lysergic acid diethylamide (1B-LSD), appears to have already reached the street market, although no seizure of this drug has yet been reported (Figure 1). The only articles published present the analysis of commercial standards, using some analytical techniques (GC–MS, NMR, LC-MS) [9], or the screening procedures for the detection of nine LSD derivatives in rat urine, including 1B-LSD, using LC–HR-MS/MS [10].

The current study reports the integrated approach used for the extraction and identification of 1B-LSD, from a blotter paper sample (labeled “1B-LSD Blotters (25 MCG)”) found in a package seized by the Swedish customs on 05/11/2018 at Arlanda airport. The chemical identification was performed using GC–MS, HR–MS, NMR and chemo-informatics tools.

## 2. Results

### 2.1. GC-MS Analysis

**Figure 2:**
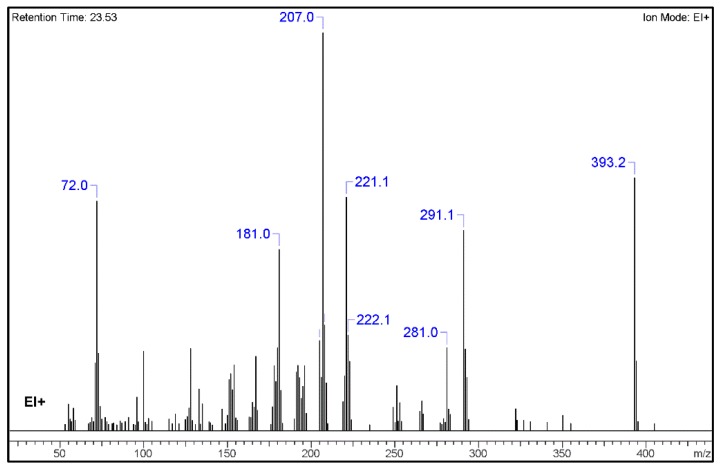
MS spectrum resulting from the GC–MS peak, identified as 1B-LSD.

From the chromatogram of the GC–MS analysis (Figure S1), it could be concluded that the matrix was quite complex. The main peak was among the last to be eluted, with a retention time of 23.5 min, and was identified as potentially 1B-LSD (see Figure 2), in agreement with the fragmentation patterns of 1P-LSD and the 1B-LSD, as reported by Brandt et al. [5,9]. The complete identification has been verified and confirmed by the results obtained from NMR and HR-MS.

The resulting identification of the most relevant fragments, as received from the chemo-informatics tool for the GC–MS analysis, are presented in Table 1. Nevertheless, it shall be highlighted that all GC–MS fragmentation patterns were consistent with those reported by Brandt et al. [9].

### 2.2. UHPLC–HR-MS Experiment

**Figure 3:**
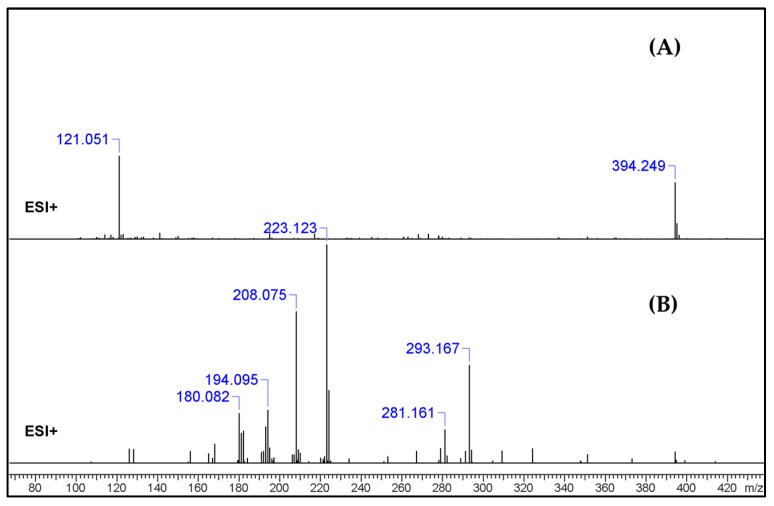
U(H)PLC–qTOF-MS (**A**) full-scan MS (**B**) untargeted MS^2^ spectrum of *m*/*z* 394.249 ([M + H]^+^) of the extract.

The full-scan UHPLC–qTOF-MS analysis (positive mode) of the blotter paper methanol extract revealed the TIC of a relatively complex mixture, in accordance with what had been observed in the GC–MS analysis (Figure S2). The extraction of the ion chromatogram (XIC) with the [M + H]^+^ of 1B-LSD (*m*/*z* 394.249), as received from the LC–qTOF-MS analysis, revealed a clear and significant peak. The analytical system allowed for a chromatographic separation to be performed whilst producing untargeted (all-ion mode) MS^2^ spectra (Figure 3).

**Figure 4:**
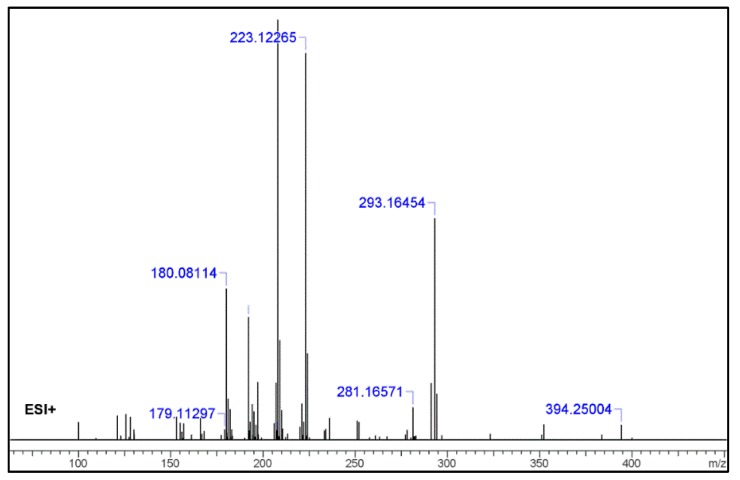
MS^2^ spectrum from Infusion to Orbitrap MS of the extract [selected molecular ion *m*/*z* was 394.25004 assigned to the protonated molecular mass [M + H]^+^.

For the Orbitrap-MS experiments, the sample extract was directly infused into the system for analysis. Full-scan MS was performed initially, with a multitude of ions being found, as the blotter paper methanol extract, containing several components, was injected directly. In fact, the lack of a chromatographic technique coupled to the Orbitrap, increased the complexity of the results. However, the previous results, indicating the possible presence of 1B-LSD, allowed an oriented extraction of its protonated molecular ion [M + H]^+^, which was found and confirmed as m/z 394.25004. After this analysis, the ion was isolated and fragmented successively (MS^2^, MS^3^ and MS^4^), and Figure 4 shows the fragmentation spectrum of this ion (MS^2^).

Some of the fragments identified with the Orbitrap-MS analysis are also consistent with those of the GC–MS, even if different ionization modes were used (ESI+ vs. EI+). All the identification was based on spectral data (Agilent MassHunter), the literature and the application of chemo-informatics tools (ACD/Labs Spectrus Processor and MS Fragmenter), a software platform that allows the prediction of fragmentation and the comparison of experimental with theoretical data. The resulting identification of the most relevant fragments, as received from the chemo-informatics tool for the Orbitrap-MS, are presented in Table 2. All results were consistent with both the qTOF-MS fragmentation and the fragmentation patterns of 1B-LSD, as reported by Brandt et al. [9].

### 2.3. NMR

**Figure 5:**
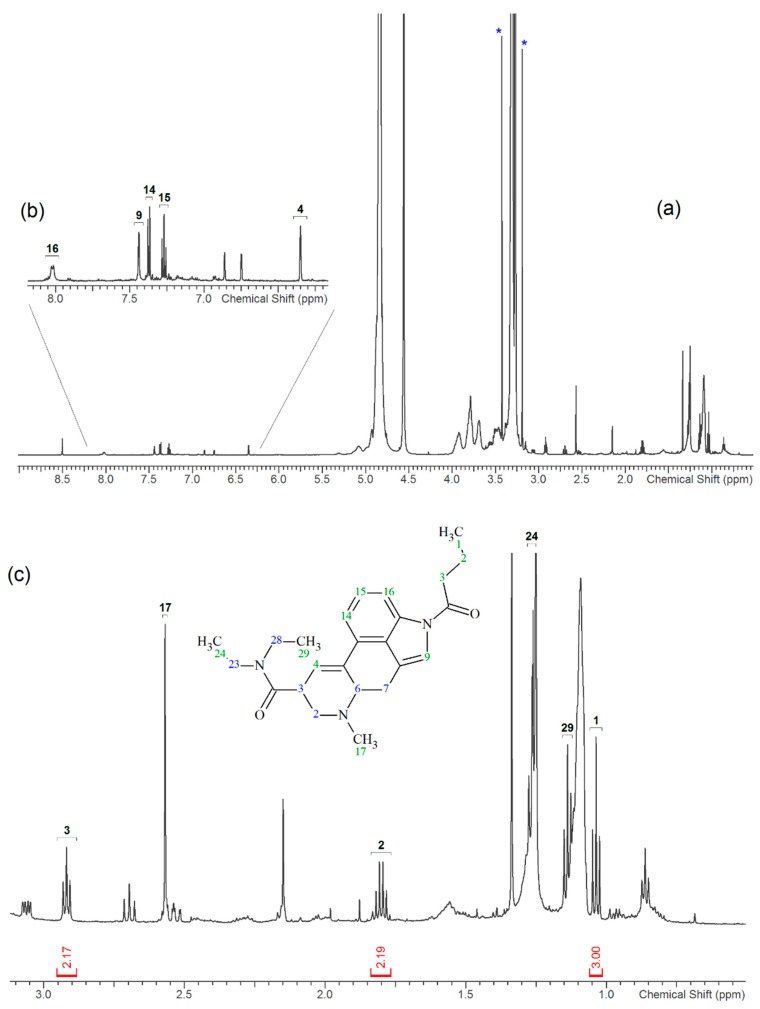
^1^H NMR spectrum 1B-LSD (**a**) full spectrum, (**b**) expansion of the aromatic region (**c**) expansion of the aliphatic region showing the region of interest for the identification of the 1-butyl group. The peaks labelled with * at 3.1 and 3.4 ppm correspond to the ^13^C satellite signals of the residual protonated NMR solvent (methanol-d4 ≥ 99.8 atom % D).

Despite the fact that the sample aliquot was so small (the amount of LSD in a blotter is usually in the range 30–100 μg), the ^1^H-NMR spectrum revealed a complex mixture (Figure 5) in which many signals could be identified as typical of LSD-like substances, and which share the same backbone structure. Nevertheless, the most noteworthy signals were those which allowed the identification of 1B-LSD in this mixture, i.e., those assigned to the butyl (CH_3_ and CH_2_) part of the molecule. On the other hand, a direct 1D ^13^C NMR measurement was not possible due to the reduced quantity of the sample and the presence of relatively large quantities of other interfering substances extracted from the original matrix. The bond connection between the butyl proton resonances was confirmed with the 2D DQF-COSY experiment (Figure S4). The 2D HSQC confirmed the presence of the methyl and methylene groups and HMBC showed the connection of the methylene groups with a signal of a quaternary carbon at 171.6 ppm in line with the C=O reported by Brandt et al. [9]. All our NMR results are consistent with those for 1B-LSD as reported by Brandt et al. [9].

## 3. Discussion

### 3.1. GC–MS and HR-MS

From the GC–MS spectrum (Figure 1), the 1B-LSD molecular ion (*m*/*z* 393) could be identified, as well as some characteristic clusters of these types of substances. More precisely, the clusters at *m*/*z* 293, *m*/*z* 292 and *m*/*z* 291, were consistent with the additional CH_2_ substituent of 1B-LSD compared to that reported by Brandt et al. [9], when compared with 1P-LSD, which presents a fragment cluster at *m*/*z* 277, *m*/*z* 278 and *m*/*z* 279, as also previously reported [5]. In the case of LSD, with no substituent at the indole nitrogen, this fragment cluster of ions is shifted towards the cluster at *m*/*z* 221, *m*/*z* 222 and *m*/*z* 223 [5,9]. With the exception of *m*/*z* 322, which represents the loss of the acyl radical from 1B-LSD, the remaining three species might have represented the butyl-substituted counterparts of those species, previously described as LSD, detected at *m*/*z* 280 (retro-Diels Alder), *m*/*z* 223 and *m*/*z* 196 (loss of *N*,*N*-diethylacrylamide). A neutral loss of *N*,*N*-diethylformamide from the M•^+^ might have led to the formation of the *m*/*z* 291 ion [5]. The EI+ characterization of 1B-LSD, 1P-LSD and LSD, presented by Brandt and other authors, was perfectly consistent with the results of the present study, and allows the unequivocal identification of 1B-LSD [5,6,7,8,9].

In the case of UHPLC–qTOF-MS analysis, the extraction of the ion chromatogram (XIC) with the [M + H]^+^ of 1B-LSD (*m*/*z* 394.249) revealed a clear and abundant peak. From the respective MS^2^ fragmentation pattern, five abundant ions were identified which related to the suspected molecular structure, namely the *m*/*z* 293.165 (loss of [(diethylamino)methylidyne]oxidanium ion), 223.124 (loss of but-1-en-1-one), 208.075 (loss of a methyl), 192.091 (loss of methylamine) and 180.081 (internal rearrangement of tetrahydropyridine with loss of a methyl). The obtained results were in accordance with previously reported works on the ESI+ analysis of 1B-LSD itself, and also 1P-LSD [5,9,11,12,13,14].

Regarding the Orbitrap-MS, the MS^2^ fragmentation pattern was in full accordance with the qTOF-MS^2^ results, with the four most abundant ions being *m*/*z* 293.16454 (loss of [(diethylamino)methylidyne]oxidanium ion), 223.12265 (loss of but-1-en-1-one), 208.07528 (loss of a methyl) and fragments 180.08114 and 181.08117 (internal rearrangement of tetrahydropyridine with loss of a methyl). Once again, these fragments are typical of 1B-LSD and 1-P-LSD [5,9,11,12,13,14].

For both the qTOF and Orbitrap-MS analysis, some of the fragments identified with the Orbitrap-MS analysis were also consistent with those of the GC–MS analysis, even though different ionization modes were used (ESI+ vs. EI+).

### 3.2. NMR

Despite the fact that the sample aliquot was so small, the ^1^H-NMR spectrum still revealed the identity of a complex mixture (Figure 4) in which many signals could be identified (like protons 14 to 16 of the aromatic ring) as typical of LSD-like substances which share the same backbone structure. However, the signals that are most noteworthy are those which allow the identification of 1B-LSD in this mixture, i.e., those assigned to the butyl (CH_3_ and CH_2_) part of the molecule with δH (MeOD-d_4_): 1.09 ppm [4] (3H, t, J = 7.4 Hz), 1.86 ppm [3] (2H, m, J = 7.4 Hz) and 2.98 ppm [2] (2H, t, J = 7.4 Hz). These results are consistent with a recent study by Brandt et al. on this substance [9].

On the other hand, the direct 1D ^13^C NMR measurement was not possible due to the reduced quantity of the sample and the presence of relatively large quantities of other substances extracted from the original matrix. However, with 2D HSQC, it was possible to confirm the presence of the methyl and methylene groups. The bond connections between the protons in this butyl group were confirmed by the 2D DQF-COSY. Furthermore, the long-range ^1^H-^13^C HMBC showed the correlation of these two methylene groups with a C=O at 171.6 ppm. These results are consistent with a recent work of Brandt et al. on this substance [9].

## 4. Materials and Methods

### 4.1. Chemical and Reagents

All solvents for the NMR, HR-MS/MS and GC–MS, were obtained from Sigma-Aldrich (Steinheim, Germany) and all LC-MS solvents were ChromaSolv grade, obtained from Fluka Analytical. Ultrapure water (18.2 MΩ) was obtained from a Milli-Q system (Millipore, Bedford, MA, USA).

### 4.2. Seized Blotter Sample

A plastic bag containing one blotter paper labeled as “1B-LSD Blotters 25 MCG” was seized at Stockholm Arlanda Airport, Sweden, together with one plastic bag containing 10 flualprazolam tablets and one plastic bag containing 0.5 g of 2-fluorodeschloroketamine (both bags were correctly labelled). No sub-sample was available for any of the seized materials.

### 4.3. Sample Preparation

The material was extracted at the Swedish Customs Laboratory with 0.5 mL of MeOH, followed by filtration. After GC–MS analysis, aliquots of the samples were shipped to the JRC for further analysis. A direct injection of the methanol sample was carried out for the UHPLC–qTOF-MS analysis, while for the HR-Orbitrap-MS, the extract was diluted 1:50 with acetonitrile.

### 4.4. Instrumental Analysis

#### 4.4.1. GC–MS

An Agilent 7890 Gas Chromatograph equipped with a 5977 MS (Agilent, Santa Clara, CA, USA) was used for the analysis of the sample. The GC-column was a HP5-MS UI (Agilent Technologies, 30 m, 0.250 mm, 0.25 mm) with helium as the carrier gas, at a flow of 0.8 mL/min. An initial oven temperature of 100 °C was set with a 5 min isothermal period followed by heating up to 300 °C at a rate of 30 °C/min and held for 13 min. The total run time was 25 min. The injection volume was 1 µL in split mode (20:1), and the injector temperature was set at 250 °C. The GC–MS operated at scan mode *m*/*z* ranging from 30–550. GC–MS analysis was performed by the Swedish Customs Laboratory, as well as at the JRC. Data have been processed with an ACD/labs spectrus processor.

#### 4.4.2. NMR

For the acquisition of a ^1^H NMR spectrum, the remaining methanol extract was mixed with deuterated methanol MeOD-d_4_ (up to a final volume of 600 μL), which was used as an internal lock and chemical shift reference at δH = 3.3 ppm. The ^1^H NMR experiments were performed at 300 K on a Bruker (Rheinstetten, Germany) spectrometer Avance III HD 600 (nominal proton frequency 600.13 MHz) equipped with a 5 mm QCI cryo-probe (^1^H, ^13^C, ^15^N and ^19^F). The ^1^H and ^13^C chemical shifts are expressed in ppm, referenced to the proton signal of the MeOD-d_4_ (3.3 ppm for ^1^H and 47.6 for ^13^C). Compounds were characterized by one-dimensional ^1^H, as well as two-dimensional ^1^H/^1^H COSY, ^1^H/^1^H TOCSY, ^1^H/^13^C HSQC, ^1^H/^13^C HMBC and ^1^H/^15^N HMBC experiments.

#### 4.4.3. HR-MS/MS

For UHPLC–qTOF-MS analysis, a UHPLC system (Agilent 1290) with a quadrupole Time-Of-Flight (TOF) mass spectrometer (Agilent 6540 UHD Accurate-Mass, Agilent, Waldbronn, Germany), using an ESI interface, operating in positive ionization mode with a 4 kV capillary voltage, was used. The source operated at 325 °C and nitrogen was used as both the drying (40 psi) and nebulizing gas (10 L min^-1^). The injection volume was 5 μL. The TOF-MS detector was set to acquire MS data over an *m*/*z* range of 100–1600. All-Ions MS/MS experiments were performed to screen and quantify constituents of the sample in a single analysis. Under the aforementioned conditions, fragmentation collision energies ranged from 5–60 eV.

The analytical column was a Waters BEH C18 100 × 2.1 mm, 1.7 μm particle size (Waters, Milford, MA, USA), temperature-controlled at 40 °C. The mobile phase consisted of water with 0.1% formic acid (A) and methanol with 0.1% formic acid (B) at a flow rate of 200 μL min^−1^. The gradient program changed linearly from 50% to 95% (B) in 25 min, followed by an isocratic elution for 4 min. An equilibration time of 1 min was set for the mobile phase to reach initial conditions again.

For Direct infusion Orbitrap-MS, and in correlation with the UHPLC–qTOF-MS analysis, additional measurements were performed using an Orbitrap MS system, to confirm the molar mass (M) and the mass fragmentation patterns of the suspected NPS. This analysis was performed in positive-ion mode with Electro Spray Ionization (ESI) using a Thermo LTQ Orbitrap MS (Thermo Scientific, Bremen, Germany), and operated with mass resolution of 140,000 at *m*/*z* 200. The sample was infused at a flow rate of 5 μL/min on the system.

### 4.5. Subsection Chemoinformatics Tools

The ACD/Labs platform (ACD/Labs, Toronto, Canada) was used in combination with Agilent’s MassHunter (Agilent Technologies) and XCalibur (Thermo Scientific) for the assessment and evaluation of the obtained data, including all the obtained chromatographic and MS data. This platform allows the confirmation and checking of the consistency of any suggested chemical structures with NMR experimental data, as well as assigning experimental spectra to structures, enabling the creation of a central, fully searchable repository of the assigned NMR spectra. The software was also used to project fragmentation paths, by comparing experimental MS and MS/MS data with theoretical data.

## 5. Conclusions

The identification of NPS in seized samples still remains a challenge for many laboratories. In cases where a novel unreported substance is found, or when a sample is seized, either in very small amounts or in a complex mixture, routine analytical techniques are often not sufficient. In these cases, an analytical workflow that combines hyphenated techniques with HR-MS, NMR and chemo-informatics tools is the most effective approach to identify and/or confirm the presence of an NPS with sufficient precision.

By following such an analytical workflow, it was possible, in this study, to identify 1B-LSD in the methanol extract of a single blotter paper by using four different techniques and chemo-informatics tools. Although previously characterized at analytical-standard levels, it is the first time that this substance was found and identified in samples circulating in the street market. This work can also be seen as proof that scientific cooperation between modern forensic laboratories can lead to the correct and reliable identification of NPS, even if present in trace amounts.

## Figures and Tables

**Table 1:** GC–MS-identified fragments for 1B-LSD (ACD/Spectrus Processor 2017.2.1).

No.	Fragment New Structure	Formula	Label	*m*/*z* Exp. ^1^	RI Exp. (%) ^2^
1	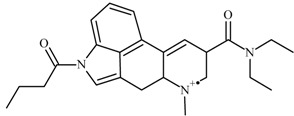	C24H31N3O2(+)	M	393.2	100.0
2	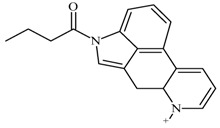	C18H15Ν2O2(+)	M-C6H16N	291.1	79.3
3	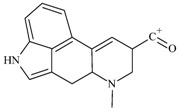	C16H15N2O(+)	M-C8H16NO	251.1	15.5
4	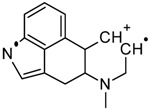	C15H13N2(+)	M-C9H18NO2	221.1	58.665
5	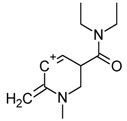	C12H19N2O(+)	M-C12H12NO	207.0	69.3
6	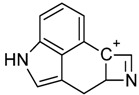	C12H9N2(+)	M-C12H11NO	181.0	45.6
7	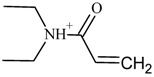	C7H14NO(+)	M-C17H20N2O	128.1	20.6
8	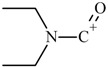	C5H10NO(+)	M-C19H21N2O	100.0	31.4
9	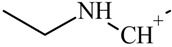	C4H10N(+)	M-C20H21N2O2	72.0	90.8

^1^*m*/*z* Exp.: experimental *m*/*z*; ^2^ RI Exp. (%): Relative Intensity (%) of experimental *m*/*z*.

**Table 2:** Orbitrap-MS-identified fragments for 1B-LSD (ACD/Spectrus Processor 2017.2.1).

No.	Fragment New Structure	Formula	Label	*m*/*z* Exp. ^1^	RI Exp. (%) ^2^
1	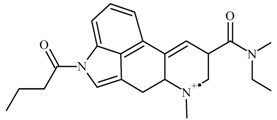	C24H31N3O2(+)	M	394.24890	3.5
2	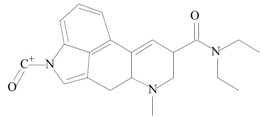	C21H26N3O2(+)	M + H-C3H6	352.20195	3.6
3	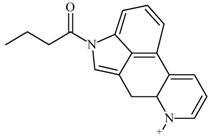	C19H21N2O(+)	M + H-C5H11NO	293.16454	52.7
4	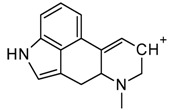	C15H15N2(+)	M-C9H16NO2	223.12265	27.5
5	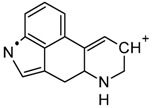	C12H20N2O(+)	M-C12H12NO	208.07528	69.3
6	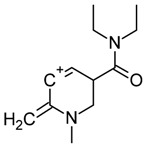	C12H19N2O(+)	M + H-C12H13NO	207.14919	13.5
7	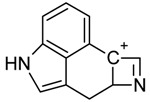	C12H8N2(+)	M-C12H11NO	180.06820	36.0
8	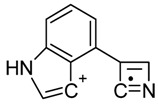	C11H6N2(+)	M-C11H9NO	166.05255	5.1
9	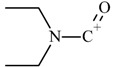	C5H10NO(+)	M-C19H22N2O	100.07569	4.5

^1^*m*/*z* Exp.: experimental *m*/*z*; ^2^ RI Exp. (%): Relative Intensity (%) of experimental *m*/*z*.

## Supplementary Materials

The following are available online, Figure S1: GC chromatogram analysis (Full scan mode) of the blotter paper methanol extract, Figure S2: UHPLC-qTOF-MS analysis of the blotter paper methanol extract (A) and the extracted ion chromatogram (XIC) of the 1B-LSD [M + H]^+^, Figure S3: 2D COSY NMR spectra of the blotter paper methanol extract; Figure S4: HMBC NMR spectra of the blotter paper methanol extract.
