# Supplementary material for: Identification of 1-Butyl-Lysergic Acid Diethylamide (1B-LSD) in Seized Blotter Paper Using an Integrated Workflow of Analytical Techniques and Chemo-Informatics
Source: Molecules. 2020 Feb 7;25(3):712. doi: 10.3390/molecules25030712 (PMC7037844; doi:10.3390/molecules25030712)
Supplement: Supplementary file 1 [file molecules-25-00712-s001.pdf]

Identification of 1-butyl-d-lysergic acid diethylamide (1B-LSD) in seized blotted paper using an integrated workflow of analytical techniques and chemoinformatics

Emmanouil D. Tsochatzis[1], Joao A. Lopes [1,] *, Fabiano Reniero [1], Margaret V. Holland [1], Jenny Åberg [2] and Claude Guillou [1]

Supplementary material

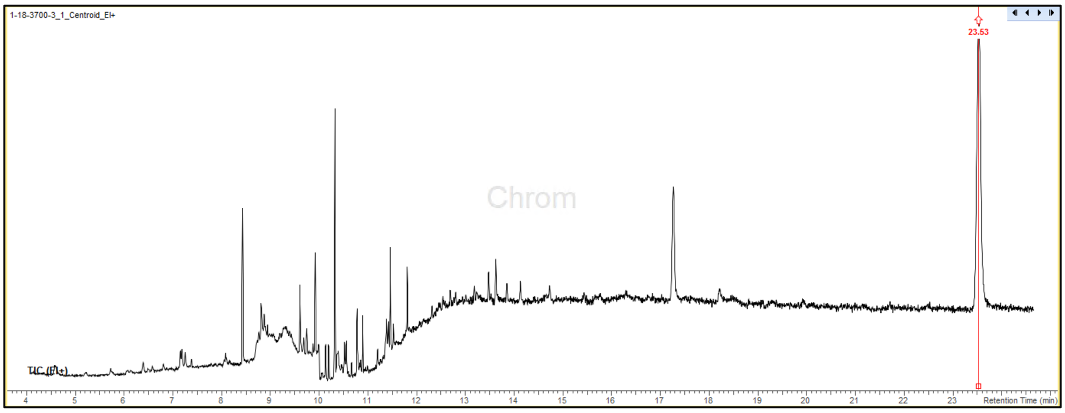

Figure S1 - GC chromatogram analysis (Full scan mode) of the the blotter paper methanol extract

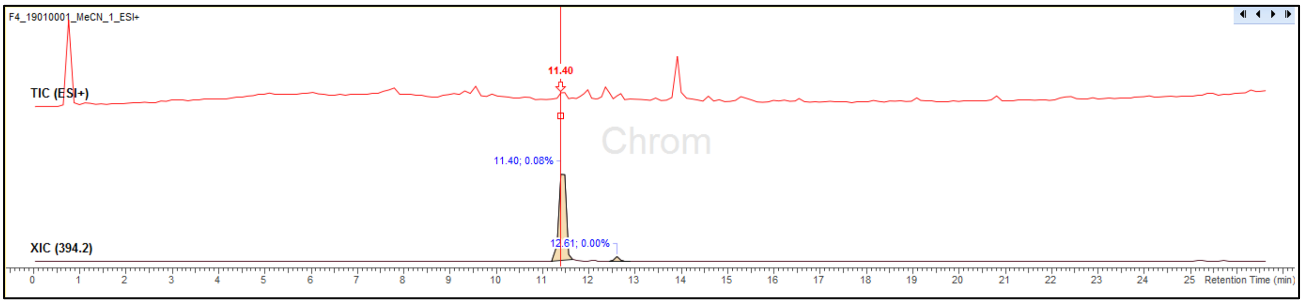

Figure S2 - UHPLC-qTOF-MS analysis of the blotter paper methanol extract (A) and the extracted ion chromatogram (XIC) of the 1B-LSD $[M+H]^+$

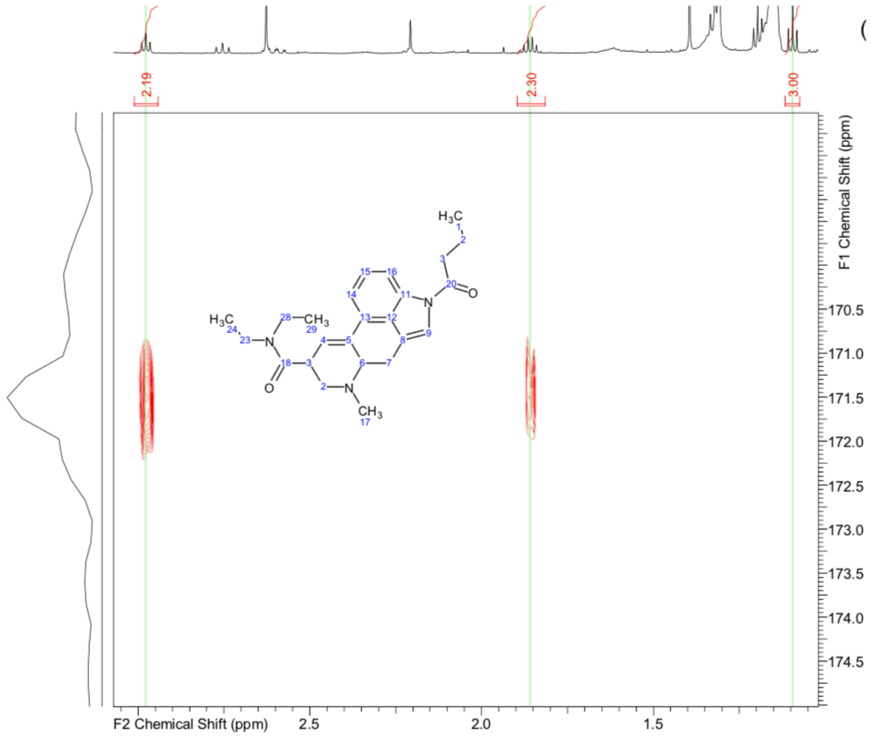

Figure S3 - HMBC NMR spectra of the blotter paper methanol extract

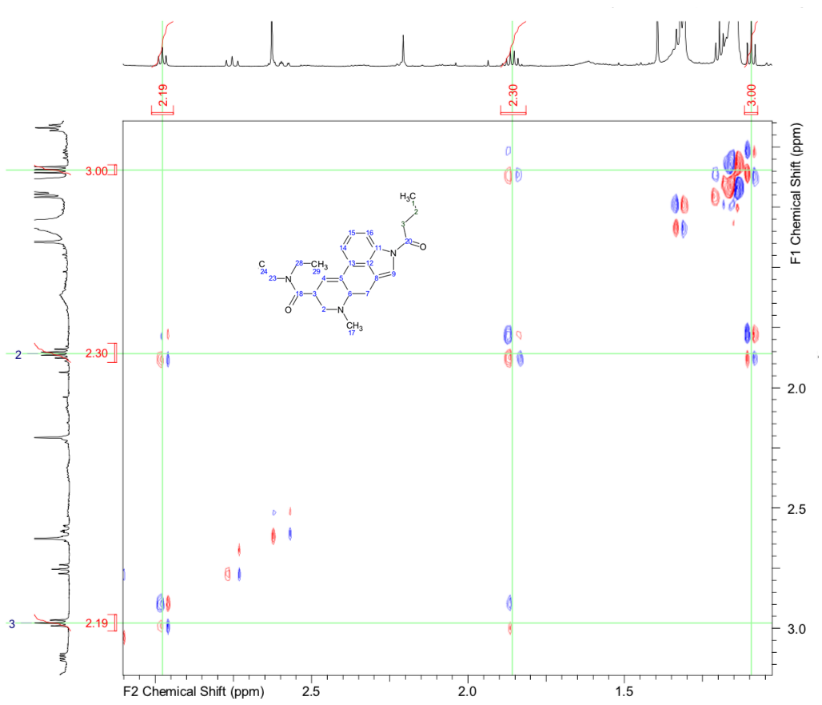

Figure S4- 2D COSY NMR spectra of the blotter paper methanol extract
